# Towards cross-Canada monitoring of the unregulated street drug supply

**DOI:** 10.1186/s12889-021-11757-x

**Published:** 2021-09-15

**Authors:** Emily Biggar, Kristi Papamihali, Pascale Leclerc, Elaine Hyshka, Brittany Graham, Marliss Taylor, Doris Payer, Bridget Maloney-Hall, Jane A. Buxton

**Affiliations:** 1 Canadian Centre on Substance Use and Addiction, 500-75 Albert Street, Ottawa, ON K1P 5E7 Canada; 2 British Columbia Centre for Disease Control, 655 West 12th Avenue, Vancouver, BC V5Z 4R4 Canada; 3 Direction régionale de santé publique, Centre intégré universitaire de santé et de services sociaux du Centre-Sud-de-l’Île-de-Montréal, 1301 rue Sherbrooke est, Montreal, QC, H2L 1M3 Canada; 4 School of Public Health, University of Alberta, 3-300 Edmonton Clinic Health Academy, 11405 87 Avenue NW, Edmonton, Alberta T6G 1C9 Canada; 5 Streetworks, Boyle Street Community Services, 10116-105 Ave, Edmonton, Alberta T5H 0K2 Canada; 6 School of Population and Public Health, University of British Columbia, 2206 E Mall, Vancouver, BC V6T 1Z3 Canada

**Keywords:** Drug monitoring, Urinalysis, Harm reduction

## Abstract

**Background:**

The well-being of people who use drugs (PWUD) continues to be threatened by substances of unknown type or quantity in the unregulated street drug supply. Current efforts to monitor the drug supply are limited in population reach and comparability. This restricts capacity to identify and develop measures that safeguard the health of PWUD. This study describes the development of a low-barrier system for monitoring the contents of drugs in the unregulated street supply. Early results for pilot sites are presented and compared across regions.

**Methods:**

The drug content monitoring system integrates a low-barrier survey and broad spectrum urine toxicology screening to compare substances expected to be consumed and those actually in the drug supply. The system prototype was developed by harm reduction pilot projects in British Columbia (BC) and Montreal with participation of PWUD. Data were collected from harm reduction supply distribution site clients in BC, Edmonton and Montreal between May 2018–March 2019. Survey and urine toxicology data were linked via anonymous codes and analyzed descriptively by region for trends in self-reported and detected use.

**Results:**

The sample consisted of 878 participants from 40 sites across 3 regions. Reported use of substances, their detection, and concordance between the two varied across regions. Methamphetamine use was reported and detected most frequently in BC (reported: 62.8%; detected: 72.2%) and Edmonton (58.3%; 68.8%). In Montreal, high concordance was also observed between reported (74.5%) and detected (86.5%) cocaine/crack use. Among those with fentanyl detected, the percentage of participants who used fentanyl unintentionally ranged from 36.1% in BC, 78.6% in Edmonton and 90.9% in Montreal.

**Conclusions:**

This study is the first to describe a feasible, scalable monitoring system for the unregulated drug supply that can contrast expected and actual drug use and compare trends across regions. The system used principles of flexibility, capacity-building and community participation in its design. Results are well-suited to meet the needs of PWUD and inform the local harm reduction services they rely on. Further standardization of the survey tool and knowledge mobilization is needed to expand the system to new jurisdictions.

## Introduction

Harms related to the unregulated street drug supply threaten the well-being of people who use drugs (PWUD). Accidental drug poisoning deaths continue to occur in alarming numbers, and have increased sharply during the COVID-19 pandemic. Between January 2016 and June 2020, 16,280 Canadians died due to accidental apparent opioid-related poisonings; record numbers were observed in at least 5 provinces and territories between April and June 2020 [1]. Deaths are largely due to contamination of the unregulated drug supply with psychoactive substances of unknown type or quantity, such as fentanyl and its analogues [1] and benzodiazepines [2]. Additional risks are posed by other modifications of drugs on the unregulated market, such as the addition of potentially harmful “cutting agents” to bulk substances [3, 4]. A better understanding of unregulated drug supply contents is needed to plan and improve responses such as observed consumption (injection or inhalation) sites, distribution of harm reduction supplies (e.g., needles, pipes), opioid agonist therapy, and harm reduction messaging.

### Existing monitoring and surveillance systems

Trends in drug use and related harms can be monitored by multiple methods of public health surveillance, including surveys, law enforcement drug seizures, coroners’ data, and drug checking services. This information is critical to detecting and responding to drug use trends with potential for harm such as intentional polysubstance use [5, 6] or unknown use of fentanyl [7]. However, these methods are often not timely, not comparable across jurisdictions, or do not reach key populations.

For example, national population-based surveys (e.g., Canadian Alcohol and Drugs Survey, Canadian Alcohol and Drug Use Monitoring Survey, Canadian Community Health Survey) provide data on self-reported use of illegal drugs but likely underestimate trends due to response and sampling bias affecting marginalized populations [8, 9]. Nationally coordinated cross-sectional surveys such as I-Track, M-Track and Y-Track (formerly Enhanced Surveillance of Canadian Street Youth or E-SYS) have improved reach to marginalized populations [10, 11], but survey cycles are infrequent. Regional cohort or cross-sectional studies of underserved populations [12–21] have improved upon the limitations described above, but methodological differences between studies (e.g., variation in reporting period of substance use) preclude comparing trends across regions. Regional studies also variably capture important subgroups of PWUD, such as those who live outside of urban centers or use drugs by non-injection means.

Other means of monitoring unregulated drug contents include analyzing samples seized by law enforcement (e.g., Health Canada’s Drug Analysis Service) [22], substances identified in urine drug screens collected in health care settings [23, 24], and substances identified by toxicology of decedents [25]. However, these methods are limited in their ability to confirm what individuals intended or expected to consume, and, in the case of toxicology data showing multiple substances, whether these were consumed at the same time or separately.

Drug checking has emerged as a quick and inexpensive method to generate data on drug contents and consumer expectations [26–28]. However, the range of detectable substances and levels of specificity and sensitivity vary among different techniques. Surrendering and handling illegal substances can also pose challenges [29].

### Towards cross-Canada monitoring of the unregulated street drug supply

The methods above contribute vital information to our understanding of substance use trends and related harms, but there remain gaps in timeliness, representativeness, and sustainability of these existing systems [30].

In 2015, the British Columbia Centre for Disease Control (BCCDC) began analyzing expected and actual drug use by combining an existing harm reduction survey with a urine norfentanyl test. This project — known as the Fentanyl Urine Screen Study (FUSS) — provided timely access to information on unintentional fentanyl use and associated behaviours (e.g., concurrent methamphetamine use) [31]. The FUSS survey tool and methodology were based on an annual survey implemented at harm reduction sites (health or social services aiming to prevent a wide range of drug-related harms) across BC from 2012 to 2015. This annual survey was identified as a feasible, low-barrier means to obtain locally-relevant drug use data from clients while also illustrating important regional differences [32]. The development, implementation and adaptation of the original survey tool has been described in detail previously [32]. In 2017, the Direction régionale de santé publique (Regional Public Health Department) of the Centre intégré universitaire de santé et de services sociaux (CIUSSS) du Centre-Sud-de-l’Île-de-Montréal (DRSP-CCSMTL) conducted a similar study that built on the FUSS methodology. Broad spectrum urine toxicology tests were employed to examine local prevalence of fentanyl and compare expected and actual substances used by participants. This study demonstrated the feasibility of using broad spectrum urine toxicology tests to examine contents of urine samples submitted by participants. Additionally, both studies identified a discrepancy between self-reported and detected use of drugs; presence of fentanyl among participants who did not report its use ranged from 73% in BC in 2015 [31] to 18% in Montreal in 2017 [33].

To scale up this pilot monitoring process, partners from the BCCDC, DRSP-CCSMTL, University of Alberta School of Public Health and Streetworks Edmonton obtained a Health Canada, Substance Use and Addiction Program grant that aimed to develop a cross-Canada surveillance system of illegal drug content using standardized tools that can be implemented across the country. The aim was to develop a system that could:

1. Monitor substance use and concordance with substances reported used
2. Respond to emerging issues regarding contamination and other drug-related harms
3. Facilitate regional comparisons
4. Evaluate interventions aimed at reducing drug-related harms; and
5. Communicate results at the local level to inform harm reduction policies and services and behavior of PWUD based on accurate information about emerging issues in the illegal street drug supply.

This paper describes the feasibility of implementing this system and preliminary indications that these goals were met.

## Methods

### Developing the pilot monitoring system

The pilot system was developed collaboratively with representatives from the BCCDC, DRSP-CCSMTL, University of Alberta and Streetworks Edmonton, and the Canadian Centre on Substance Use and Addiction (CCSA). These partners built upon the design used in the FUSS and Montreal drug testing projects described above. For this pilot system, the survey focused on past three-day drug use, history of overdose and injection drug use and basic demographics (age, gender). The three-day reporting period was chosen since it is consistent with the time frame of detection of most drugs in urine [34] and improves recall [35]. The survey consisted of “core” questions common to all sites (e.g., drugs used, experience of opioid overdose, injection drug use), and optional additional items based on local and emerging needs. All survey content was developed with input from PWUD.

In parallel with survey development, protocols were also developed for analysis of urine samples by different laboratories (LifeLabs, Ontario for BC and Edmonton sites, and Centre de toxicologie du Québec for Montreal sites). Briefly, samples were to be screened with liquid chromatography and mass spectrometry for approximately 150 drugs, including 40 opioids and several fentanyl analogues. The broad spectrum urine toxicology screen method used by LifeLabs has been described in detail previously [36]. The cost of urine toxicology ranged between 75 and 90 Canadian dollars per sample.

### Recruitment and data collection

The survey and urine sample collection were piloted between May 2018 and March 2019 at harm reduction sites in BC (May to August, 2018), Edmonton (March, 2019) and Montreal (August to September, 2018). Sites included harm reduction supply distribution services that stood alone or were integrated with supervised consumption sites or other health and social services for PWUD. Sites were recruited based primarily on site capacity (e.g., availability of staff to support data collection, washroom facilities for urine sample collection, hours of operation), as well as willingness to participate and feedback from regional harm reduction coordinators.

Participant recruitment and data collection ranged from two to four weeks. Direct service providers or PWUD involved in providing site services approached clients and invited those eligible to participate. Participants provided verbal consent, a low-barrier approach that does not require personal identifying information and is consistent with low-barrier requirements for receiving harm reduction services [32]. Participants were included if they were over the provincial age of majority and reported use of an illegal drug other than cannabis in the past 6 months (BC and Edmonton) or 3 days (Montreal). In Edmonton and Montreal, all participants completed the survey and urine sample and were offered ten dollars in compensation. In BC, the urine sample was optional, and participants were compensated with five dollars for each of the survey completion and providing a urine sample. Participating sites in BC and Edmonton were also provided five dollars per participant enrolled to remunerate the site for staff assistance with recruitment and data collection. Staff were encouraged to assist with administration of the paper-based survey at all sites, though participants wishing to self-administer were permitted to do so.

### Survey data and urine sample analysis

The completed survey and urine sample obtained from each participant were assigned matching anonymous ID codes to allow for data linkage while maintaining participant anonymity. Survey and toxicology data were received, entered, linked and analyzed by BCCDC for BC and Edmonton sites and by DRSP-CCSMTL for Montreal sites. Results were summarized by site and region, and presented to participating sites, local harm reduction coordinators and other stakeholders (e.g., harm reduction and overdose response teams, provincial ministries of health).

## Results

Data were collected from a total of 878 participants at 40 harm reduction sites across Canada. This included 27 sites (*n* = 486 participants) in BC, 1 site (*n* = 49) in Edmonton, and 12 sites (*n* = 343) in Montreal (Table 1). The median number of substances reported used by participants in the previous 3 days ranged from two (BC and Montreal) to three (Edmonton).

**Table 1:** Demographics of survey participants in three pilot sites, 2018/2019

	BC n (%^a^)	Edmonton n (%^a^)	Montreal n (%^a^)
**Number of sites**	27 (100.0)	1 (100.0)	12 (100.0)
**Participants**	486 (100.0)	49 (100.0)	343 (100.0)
**Gender**			
Male	301 (62.3)	37 (75.5)	205 (68.8)
Female	173 (35.8)	12 (24.5)	88 (29.5)
Other	9 (1.9)	0 (0.0)	5 (1.7)
**Age (median)**^b^	41	48	35–44
**Number of substances reported used**^c^**(median)**	2	3	2

^a^Percentages exclude missing data from the denominator

^b^The survey used in Montreal presented age as a categorical variable with the following age groups: 18–24, 25–34, 35–44, 45–54, 55–64, and 65+. The greatest percentage of participants were between 35 and 44 years (35%); this has been used as a proxy median value for comparison

^c^Excluding alcohol and cannabis

Urine samples were collected for 313 (64.4%), 48 (98.0%) and 341 (99.4%) participants in BC, Edmonton and Montreal, respectively. Of these, all samples had at least one substance detected, except for BC in which 309 samples (63.6%) had at least one substance detected. Reported use and detection of substances varied between regions (Table 2). Key findings are described below.

**Table 2:** Drug use among participants with urinalysis in three pilot sites, 2018/2019

	BC *N* = 309%		Edmonton *N* = 48%		Montreal *N* = 341%	
Substances	Reported	Detected	Reported	Detected	Reported	Detected
***Stimulants***						
Cocaine (powder)	20.1	–	10.4	–	40.8	–
Crack	22.7	–	35.4	–	62.4	–
Cocaine or crack^a^	29.4	42.4	39.6	27.1	74.5	86.5
Methamphetamine	62.8	72.2	58.3	68.8	14.9	43.0
Speed^b^	–	–	–	–	24.8	–
***Opioids***						
Heroin	48.5	–	20.8	–	22.7	–
Morphine	15.2	–	35.4	–	12.8	–
Heroin or morphine^c^	52.8	55.0	54.2	43.8	30.6	22.2
Fentanyl^d^	41.0	59.2	29.2	29.2	3.5	9.7
Hydromorphone	6.8	20.4	35.4	52.1	22.0	14.7
Methadone	27.0	31.4	6.3	6.3	25.9	28.7
***Depressants***						
Benzodiazepines	12.3	9.7	22.9	29.2	16.0	12.6

^a^Broad spectrum urine toxicology testing cannot distinguish between crack and cocaine

^b^The survey used in Montreal listed methamphetamine and speed separately as the content of the latter was not clear to participants or researchers. Accordingly, a measure of detected use of speed is not applicable in the context of this research

^c^Detected use of heroin or morphine includes 6-monoacetylmorphine (6-MAM; a direct metabolite of heroin), morphine, and morphine metabolites. Detected use is presented together as 6-MAM clears rapidly from urine, after which it is difficult to discern between heroin or morphine use

^d^Fentanyl and fentanyl analogues

Fentanyl use — expected, actual and unintentional — varied greatly between regions. Fentanyl was reported and detected among a smaller proportion of participants in Montreal (3.5 and 9.7% respectively) than reported and detected in other sites (41.0 and 59.2% in BC and 29.2 and 29.2% in Edmonton) (Table 2). Unintentional fentanyl use among the entire sample (i.e., participants with fentanyl detected that did not report using it) ranged from 21.4% in BC to 6.3% in Edmonton (Table 3). The proportion of participants that did not report fentanyl use among only those with fentanyl detected ranged from 90.9% in Montreal, 78.6% in Edmonton, to 36.1% in BC (data not shown).

**Table 3:** Selected outcomes among participants with urinalysis

	BC *N* = 309%	Edmonton *N* = 48%	Montreal *N* = 341%
**Unintentional fentanyl use**^a^	21.4	6.3	8.9
**Adulterants detected in urine**			
Levamisole	10.4	0.0	49.6
Lidocaine	1.0	2.1	24.6
**Experienced an opioid overdose**^b^	19.4	28.6	4.0
**Injection drug use**^c^	44.0	69.4	42.0

^a^Refers to the percentage of participants for which fentanyl was detected in urine and not reported used among the entire urinalysis sample

^b^In BC and Edmonton, participants were asked if they had experienced an accidental opioid overdose in the past six months. In Montreal, the reporting period was the past three days

^c^In BC and Edmonton, participants were asked if they had injected drugs in the past month. In Montreal, the reporting period was the past three days

Reported and detected use of methamphetamine (crystal meth) were similar within and between BC and Edmonton (58.3–72.2% of participants). In Montreal, cocaine or crack were reported used (74.5%) and detected (86.5%) most frequently.

Presence of adulterants (non-psychoactive substances whose presence is not typically expected by consumers) also ranged greatly between regions. In Montreal, levamisole and lidocaine were detected among nearly 50 and 25% of participants, respectively. Detection was most frequent among those reporting cocaine/crack use. In comparison, levamisole was only detected among 10.4% of participants in BC and not detected in Edmonton; rates of lidocaine detection were 1.0 and 2.1%, respectively.

Injection drug use was prevalent in all regions, with a high of 69.4% in Edmonton.

## Discussion: lessons learned from development of pilot system

Preliminary results of the pilot monitoring system suggest that, like one of the prototypes [32], it is acceptable to harm reduction sites and clients, can be feasibly implemented in different jurisdictions, and yields meaningful results. Furthermore, the study identified the following four important lessons:

### 1. The system can help understand local needs and inform public health programs and policy

The quantitative results presented here can help understand local trends and needs, and can justify future work to understand and address the emerging needs of PWUD. For example, stimulant use was prevalent in all regions, raising the need to better understand associated demands (e.g., for pipes and other harm reduction supplies [37]) and supports for those who use stimulants only or in combination with opioids. On a similar note, polysubstance use was another important trend. Most participants reported using two (BC, Montreal) or three (Edmonton) substances. Reported polysubstance use was consistent with previous research in similar populations [38–40]. There is a need to better understand why PWUD use multiple substances in specific patterns given accumulating evidence on risk of harms related to poor physical and mental health [41] and overdose [1, 42, 43]. Harms may be compounded by adulterants such as levamisole, a non-psychoactive substance found in cocaine or crack that poses risk of multiple serious adverse effects [2, 3, 44, 45].

Results may also be used to track trends over time. Rates of unintentional fentanyl use observed in the present study have decreased compared to earlier prototype studies. In BC, among those with fentanyl detected, only 36.1% used unintentionally in 2018 versus 72.8% in 2015 [31]. Among the entire sample in Montreal, fentanyl was detected but not reported used among 8.9% of respondents in 2018 versus 18.0% in 2017 [33]. It will be important to continue to monitor trends in intentional and unintentional fentanyl use to ensure overdose prevention efforts remain relevant and evidence-informed.

This system also provides an opportunity to examine use of prescription opioids (POs) (e.g., methadone, morphine, hydromorphone) by individuals accessing harm reduction services. Participants reporting use of POs in this study may have obtained them with a prescription or from the diverted supply in the unregulated market. The latter is preferred by some PWUD who face barriers to obtaining a prescription but wish to use opioids of predictable type and quantity [46]. To further complicate matters, apparent diverted POs in the street supply may also be illegally manufactured. Counterfeit POs often contain unexpected substances of varying potency, including novel synthetic opioids such as fentanyl and fentanyl analogues [47]. Given the interplay between PO use and harms experienced by PWUD in Canada [48], a nuanced understanding of use by clientele of harm reduction services is needed to inform interventions such as safer supply.

In addition to assessing expectations of drug contents, the survey also provides an opportunity to gather detailed contextual information about drug use. Results presented here are a small snapshot of information available from the survey, which includes, for example, reasons for using drugs alone [49], unintentional fentanyl use [36], naloxone kit access [50], acceptability of overdose monitoring applications [51], and changes in methadone formulation [52]. This information has been used to provide feedback for improvement and quality assurance of harm reduction services, and findings have helped create targeted knowledge translation approaches. For instance, naloxone kit ownership improved after identifying lower ownership among those who smoke substances [50]. Other trends currently under investigation include knowledge and understanding of the Good Samaritan Drug Overdose Act, concurrent methamphetamine and opioid use, and shifting modes of opioid use (i.e., to inhalation). This information will likely inform future harm reduction interventions that are responsive to the realities of PWUD. In the future, optional content can be further adapted to allow regions flexibility to ask questions that are of local interest, reflect the local drug use and harm reduction landscape and its emergent issues, or evaluate local interventions.

### 2. The system is low-barrier

The pilot system leveraged existing harm reduction supply distribution sites and used a low-barrier design suitable to these environments (e.g., anonymous participation). This allows the system to be administered regularly in many types of sites accessed by PWUD with respect to service delivery model (standalone or integrated harm reduction services), geography (urban, rural or remote), and capacity for data collection. In addition, unlike with methods such as drug checking, participants are not faced with the choice of surrendering drugs for testing, and staff are not required to handle illegal samples or interpret complex results. Toxicological screening also allows systematic detection of a wide range of substances across participating sites, which is not always possible with other drug checking methods (e.g., fentanyl immunoassay strips) [29].

### 3. The system can help in relationship-building between participants and harm reduction sites

The original survey tool implemented in BC led to additional positive benefits in harm reduction sites [32]. Its anonymous nature encouraged client participation, and involvement of site staff (including PWUD) in data collection provided an opportunity to engage and build positive rapport with clients. The current system preserves this reciprocal process: participants are able to describe their experiences with the unregulated street drug supply, and site staff are able to learn about these experiences. Aggregate results are then shared with clients to ensure they feel heard and that their knowledge is accurately represented. As discussed by Wallace et al. [53] in the context of drug checking services, trust is the keystone of harm reduction wherein any intervention is “implemented in a context of stigma, trauma and other risks”.

### 4. The system encourages participatory research

The study incorporated principles of participatory research, which greatly improved the relevance, reach and uptake of the pilot through relationships with networks of PWUD and harm reduction service providers. For instance, PWUD were encouraged and compensated for leading participant recruitment and data collection. In some sites, these partners also guided development of the survey tool and disseminated findings through their networks. The latter is crucial as knowledge shared within communities of PWUD can be highly influential [54]. Such participatory processes form the backbone of an effective drug content monitoring system [30] and improve the rigor, relevance and reach of results for PWUD and their communities – the “3 R’s” of participatory research [55].

### Limitations

There are several limitations to the urine toxicology screening method. Drug detection timeframes and the reporting period (past three-day use) align for most but not all substances [34]. For instance, prevalence of use may be underestimated for drugs such as fentanyl and heroin which clear rapidly from urine. It can also be difficult to determine the origin of certain detected substances (e.g., morphine and metabolized heroin-related compounds are indistinguishable). The screening method only indicates whether a substance is present but not how much. Additionally, one can examine associations between expected and unexpected substances (e.g., cocaine/crack and levamisole) but cannot conclude precisely where contamination occurred if multiple substances are reported used. For these reasons, urine toxicology screening is complementary to drug checking services that can provide detailed qualitative and quantitative information about drug contents [29]. Various monitoring methodologies may be combined to further clarify contents of the street drug supply year to year; the BC Drug Overdose and Alert Partnership is one example of this synthesis of monitoring data [23].

Availability of staff time and washrooms to facilitate urine collection were among the primary factors in site selection. Risk of selection bias is low as a systematic relationship between these factors and drug use is unlikely. While non-response rates were not available, evidence of low refusal and non-completion rates in an earlier prototype [32] and the low-barrier context of data collection suggest non-response bias would be minimal. However, findings from this pilot project may not be generalizable to all harm reduction sites and PWUD in each region.

Additional limitations relate to the reliance on anonymous self-report survey data. A small number of individuals may have participated multiple times. Any effect on results is likely minimal, especially as drug use data would be novel if contributed three or more days apart. Recall of used substances may be biased, though the chosen reporting period (past three-day use) improves recall accuracy compared to longer reporting periods [35] and is consistent with most urine drug detection timeframes [34]. Underreporting of certain self-reported behaviours due to social desirability bias may also have occurred. However, evidence that the survey tool provides an opportunity for rapport-building suggests that respondents have positive and trusting interactions with the staff or PWUD collecting data [32].

Finally, there are limits to the comparability of the three sites in this study. In this pilot phase, inclusion criteria differed slightly between regions and may have led to samples with slightly different substance use characteristics. This criterion differed due to historical eligibility for the studies upon which this system is based. In addition, some results from the pilot phase are not strictly comparable across sites due to differences in survey question reporting periods. Future phases of the project will standardize participant eligibility criteria and reporting periods to improve comparison of results across all regions.

### Next steps

In light of the results presented here, trends in unintentional or unknown substance use will be monitored closely in future iterations of this project. This need is further justified by the record-high numbers of accidental drug poisoning deaths coinciding with the COVID-19 pandemic [56, 57]. Validity of self-reported substance use may also be measured, as done recently for methamphetamine use in BC [58]. Future work may also examine how substances such as POs are obtained (e.g., with or without a prescription) to provide further context on the state of the unregulated street supply.

In the study’s next phase, partners will collaborate to standardize materials and methods and facilitate scale-up to additional sites across Canada. A “project toolkit” will be developed, including a standardized survey containing core and optional content. Adaptation of optional content according to local relevance, need, and consultation with PWUD and harm reduction stakeholders will be encouraged; question wording will be kept as similar as possible to maintain comparability. The “project toolkit” will also contain guidelines based on best practices in implementation, data analysis and result dissemination to expedite these processes for new sites, particularly those with few resources available for research. This form of knowledge translation prioritizes capacity-building and system sustainability. Following scale-up to additional regions, results from all participating sites will be analyzed to form a national picture on key drug use trends.

## Conclusion

The pilot project described here was successful in yielding meaningful results with high feasibility and acceptability of implementation. This was achieved by creating an adaptable, low-barrier methodology for monitoring contents of drugs on the unregulated market that emphasizes participation of PWUD. For these reasons, this project may be easily scaled to other jurisdictions to address gaps in knowledge about drug-related harms. Further scale-up would improve capacity for monitoring trends in drug contents, drug use behaviours and preferences across regions. This may be achieved through continued system standardization and knowledge translation on implementation processes.

## Data Availability

The datasets generated and/or analysed during the current study are not publicly available due them containing information that could compromise research participant privacy or consent, but are available from the corresponding author on reasonable request.

## Abbreviations

PWUD: People who use drugs
BC: British Columbia
BCCDC: British Columbia Centre for Disease Control
FUSS: Fentanyl Urine Screen Study
DRSP-CCSMTL: Direction régionale de santé publique, Centre intégré universitaire de santé et de services sociaux du Centre-Sud-de-l’Île-de-Montréal
CCSA: Canadian Centre on Substance Use and Addiction
